# Understanding the Ultra-Rare Disease Autosomal Dominant Leukodystrophy: an Updated Review on Morpho-Functional Alterations Found in Experimental Models

**DOI:** 10.1007/s12035-023-03461-1

**Published:** 2023-07-14

**Authors:** Irene Neri, Giulia Ramazzotti, Sara Mongiorgi, Isabella Rusciano, Marianna Bugiani, Luciano Conti, Margot Cousin, Elisa Giorgio, Quasar S. Padiath, Giovanna Vaula, Pietro Cortelli, Lucia Manzoli, Stefano Ratti

**Affiliations:** 1 Cellular Signalling Laboratory, Anatomy Centre, Department of Biomedical and Neuromotor Sciences (DIBINEM), University of Bologna, 40126 Bologna, Italy; 2 Department of Pathology, Amsterdam University Medical Centers, Vrije Universiteit and Amsterdam Neuroscience, 1105 Amsterdam, The Netherlands; 3 Department of Cellular, Computational, and Integrative Biology (CIBIO), Università Degli Studi Di Trento, 38123 Povo-Trento, Italy; 4 Center for Individualized Medicine and Department of Clinical Genomics, Mayo Clinic, Rochester, MN 55905 USA; 5 Department of Molecular Medicine, University of Pavia, 27100 Pavia, Italy; 6 Medical Genetics Unit, IRCCS Mondino Foundation, 27100 Pavia, Italy; 7 Department of Human Genetics, Graduate School of Public Health, University of Pittsburgh, Pittsburgh, PA 15261 USA; 8 Department of Neuroscience, Azienda Ospedaliera-Universitaria Città della Salute e della Scienza, 10126 Turin, Italy; 9 IRCCS, Istituto Di Scienze Neurologiche Di Bologna, 40139 Bologna, Italy; 10 Department of Biomedical and Neuromotor Sciences (DIBINEM), University of Bologna, 40126 Bologna, Italy

**Keywords:** ADLD, Cellular Morphology, Cellular Signaling, Lamin B1, Rare Diseases, Neurodegenerative Diseases, Demyelination

## Abstract

Autosomal dominant leukodystrophy (ADLD) is an ultra-rare, slowly progressive, and fatal neurodegenerative disorder associated with the loss of white matter in the central nervous system (CNS). Several years after its first clinical description, ADLD was found to be caused by coding and non-coding variants in the *LMNB1* gene that cause its overexpression in at least the brain of patients. *LMNB1* encodes for Lamin B1, a protein of the nuclear lamina. Lamin B1 regulates many cellular processes such as DNA replication, chromatin organization, and senescence. However, its functions have not been fully characterized yet. Nevertheless, Lamin B1 together with the other lamins that constitute the nuclear lamina has firstly the key role of maintaining the nuclear structure. Being the nucleus a dynamic system subject to both biochemical and mechanical regulation, it is conceivable that changes to its structural homeostasis might translate into functional alterations. Under this light, this review aims at describing the pieces of evidence that to date have been obtained regarding the effects of *LMNB1* overexpression on cellular morphology and functionality. Moreover, we suggest that further investigation on ADLD morpho-functional consequences is essential to better understand this complex disease and, possibly, other neurological disorders affecting CNS myelination.

**Graphical Abstract:**

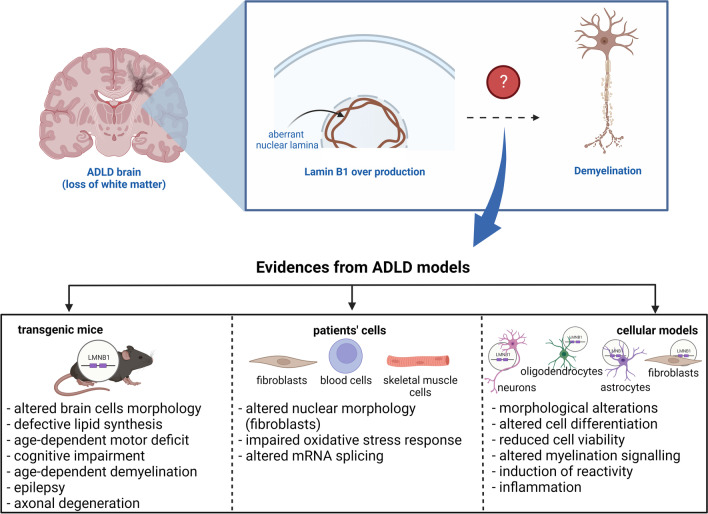

## Background

Autosomal dominant leukodystrophy (ADLD) is an ultra-rare, slowly progressive, and fatal neurodegenerative disorder associated with the loss of white matter in the central nervous system (CNS) [1, 2]. For many years, ADLD has been misdiagnosed as multiple sclerosis (MS), a more frequent pathology also affecting CNS white matter [3]. Indeed, ADLD and MS share some common features, i.e. autonomic symptoms and prominent white matter loss in the CNS [4]. However, while MS is an inflammatory, auto-immune disorder, ADLD was found to have a genetic basis, with alterations in the *LMNB1* gene being its molecular cause [1], and no sign of brain inflammation has ever been found in ADLD patients. Duplications encompassing the *LMNB1* gene or non-coding deletions at the *LMNB1* locus have been described, both leading to increased pathologic Lamin B1 protein over production. More than 30 affected families around the world have been reported to date. However, ADLD diagnosis is not always straightforward, therefore exact prevalence data are still lacking [5]. Involving the loss of white matter linked to genetic alterations, ADLD is thought to belong to the family of leukodystrophies, monogenic disorders of the white matter due to defects in any of its structural components [6]. Nevertheless, while most leukodystrophies display an early onset during infancy and childhood, ADLD clinical symptoms appear during the fourth-fifth decade of life, most commonly with autonomic dysfunction as the first evidence [5, 7, 8]. Autonomic dysfunction is often followed by pyramidal and cerebellar involvement in months to years. Even though cognitive function is usually preserved, dementia and psychiatric problems may occur as late manifestations [5, 8]. It has been reported that patients usually survive 10–20 years after onset [9, 10], but their quality of life is indeed strongly impacted. In fact, the autonomic dysfunction symptoms include severe limiting conditions such as neurogenic orthostatic hypotension and bladder dysfunction, while the pyramidal and cerebellar involvement leads to ataxia, spasticity, and intention tremor [7]. Surprisingly, autonomic disfunction is affecting only the noradrenergic control of cardiovascular system and bladder, which is physiologically performed by non-myelinated fibers [11]. Therefore, the autonomic system involvement might not be directly liked to myelin loss, but to other still unknown Lamin B1 overexpression-related pathogenic effects. Noteworthy, specific brain and spinal cord magnetic resonance imaging (MRI) findings are found in ADLD patients [5], including diffuse, confluent, and symmetrical white matter signal abnormalities mainly involving the fronto-parietal and cerebellar regions [12–16]. In this regard, the ADLD demyelination pattern is different from the MS demyelination pattern, the latter being asymmetrical [17, 18]. The spinal cord is also involved, with signal intensity changes suggesting white matter abnormalities [19]. Despite the progress made in ADLD clinical characterization and diagnosis, its pathological mechanisms remain unclear. The lack of knowledge on ADLD pathological process determines the current absence of a therapeutic protocol. Any existing treatment is directed towards the symptoms’ management; for example, orthostatic hypotension can be minimized by pharmacologic intervention, compression stockings, physical therapy, and increased dietary salt. Therefore, the role of research in understanding ADLD pathological mechanisms is pivotal. Indeed, ever since the discovery of ADLD genetic origin it has been clear that Lamin B1 overexpression must somehow cause progressive demyelination selectively in the CNS. However, how this cause-effect process takes place is yet to be clarified.

## ADLD Genetic Aspects

**Fig. 1:**
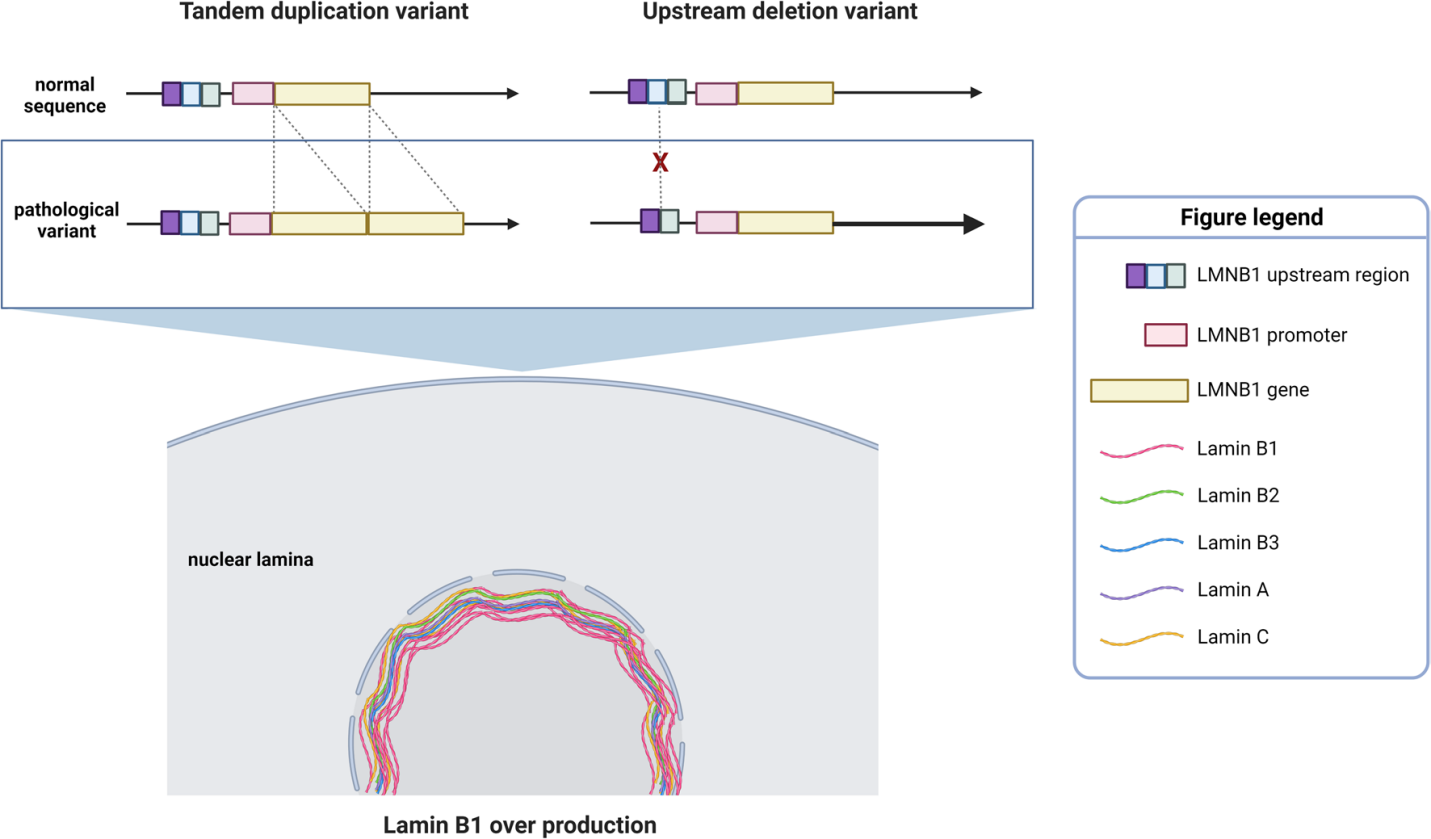
Schematic representation of ADLD genetic aspects. ADLD is caused by tandem duplications of the *LMNB1* gene or by deletions of part of the region located upstream of its promoter. In the tandem duplication variant, an extra copy of *LMNB1* is transcripted, while in the upstream deletion variant, it appears that gene expression is enhanced (indicated by the thicker arrow). Therefore, both genetic alterations lead to the over production of the protein Lamin B1, creating a quantitative disproportion between it and the other lamins that constitute the nuclear lamina. Image created with BioRender.com.

The *LMNB1* gene is located on chromosome 5q23.2 and encodes for Lamin B1. Lamin B1 belongs to the family of lamins, type V intermediate filament proteins that compose the nuclear lamina. The nuclear lamina is a fibrous network adjacent to the inner nuclear envelope [20]. Humans possess two main types of lamins: A type lamins, Lamin A and Lamin C both encoded by the *LMNA* gene, and the B type lamins Lamin B1, B2, and B3; Lamin B1 is encoded by *LMNB1* while Lamin B2 and B3 are different splicing forms of the *LMNB2* gene [21]. The nuclear lamina was first described as a structural component of the nucleus with the role of maintaining its architecture, but further studies pointed out that it is also involved in various cellular processes such as the movement of macromolecules in and out of the nucleus, cellular proliferation, senescence, ageing, DNA replication, and chromatin organization [22, 23]. In particular, it has been shown that the nuclear lamina provides anchoring sites for chromatin domains enriched in silenced genes [24, 25]. Indeed, the nuclear lamina is now considered a proper active interface between the nucleus and the cytoplasm, and its role in regulating several cellular processes is universally recognized. However, how lamins precisely act in these processes is still unclear. Strikingly, many diseases are associated with lamins alterations, especially those caused by variants in *LMNA* such as progeria and hereditary cardiomyopathy [26, 27]. ADLD has been considered the only disease associated with *LMNB1* for many years [7, 28]. Only recently *LMNB1* alterations have also been reported in other diseases such as microcephaly and neural tube closure defects [29]. However, while in these disorders the mutations are located inside the *LMNB1* coding region [30, 31], ADLD is caused by *LMNB1* duplications or, more rarely, by deletions located upstream of its promoter [1, 10, 32, 33]. *LMNB1* duplication cases and upstream deletions cases lead to a common pathological mechanism, *i.e.*, the overexpression of *LMNB1*, which results in exaggerated Lamin B1 protein production [7] (Fig. 1). In particular, in the tandem duplication variant, an extra copy of *LMNB1* is transcripted, while in the upstream deletion variant, it appears that gene expression is enhanced [7]. In ADLD, *LMNB1* overexpression is associated with a severe demyelinating phenotype [1]. Notably, ADLD is currently the only known disease in which *LMNB1* overexpression has been linked to demyelination. However, how *LMNB1* overexpression precisely leads to demyelination remains unclear.

## ADLD Experimental Models

To date, ADLD studies have been conducted using different experimental models, including transgenic mice [34–36], patients’ fibroblasts [37–40], skeletal muscle fibers [40], blood cells [37], and murine or human brain primary cells/cell lines in which *LMNB1* overexpression was induced using lentiviral vectors [38, 39, 41–43] (Table 1). Two ADLD mouse models have been created and studied: *LMNB1*^BAC^ transgenic mice and PLP-*LMNB1*^Tg^ transgenic mice (also referred to as PLP-FLAG-*LMNB1* transgenic mice or PLP-*LMNB1* transgenic mice) [34]. The first mouse model carries additional copies of the entire murine wild-type *LMNB1*, while the second overexpresses a FLAG-tagged human Lamin B1 selectively in oligodendrocytes. Notably, overexpression of the FLAG-tagged human Lamin B1 selectively in astrocytes and neurons did not cause age-dependent motor deficits and therefore these mouse models have been abandoned [34]. *LMNB1*^BAC^ transgenic mice show cognitive impairment, age-dependent motor deficits, epilepsy, aberrant myelin formation, axonal degeneration, and age-dependent demyelination [34], while PLP-*LMNB1*^Tg^ mice present aberrant myelin formation, axonal degeneration, age-dependent motor deficits, epilepsy and demyelination [34], and age-dependent degeneration of the spinal cord white matter [35]. On the other hand, studies performed on ex vivo and in vitro models provided several pieces of information about the morphological and functional consequences of Lamin B1 overproduction at the cellular level. Regarding the ex vivo models, several observations have been made. Patients’ fibroblasts displayed nuclear morphological alterations [38, 39], aberrant mRNA splicing [37], inflammation [38], and oxidative stress [38–40]. Patients’ skeletal muscle fibers showed oxidative stress [40]. Patients’ blood cells showed aberrant mRNA splicing [37]. Interesting evidence has also been obtained from ADLD in vitro models. Observations made on *LMNB1* overexpressing oligodendrocytes have been controversial: while some studies reported the presence of nuclear morphological alterations [41], defects on lipid synthesis [34, 35], and aberrant cell differentiation [41], a more recent study reported that these cells do not display significant morphological or functional alterations [38]. Studies performed on *LMNB1* overexpressing astrocytes reported the presence of important morphological alterations [12, 15, 38, 41], aberrant cellular signaling [38], and reduced cell viability and activation of the reactive status [42]. The analysis of *LMNB1* overexpressing neurons displayed important morphological alterations [39, 41, 43]. Lastly, LMNB1 overexpressing fibroblasts displayed nuclear morphological alterations and inflammation [38]. It is necessary to point out that each of these models has limitations. Indeed, neither of the two in vivo models is able to fully recapitulate the clinical phenotype observed in ADLD patients, since they do not display the autonomic symptoms that are characteristic of ADLD initial stages [36]. On the other hand, the studies performed on patient-derived cells might have similar limitations, since they have not been performed on brain cells. Finally, the studies performed on cell cultures have the limitation of not providing information on the late age dependence or systemic aspects of the disease. Under this light, much work has still to be done. Nevertheless, with the aim of stimulating further research, it is still important to discuss the evidence obtained so far. Indeed, *LMNB1* overexpression seems to affect both cellular morphology and function.

**Table 1:** ADLD experimental models

Model type	Experimental model	Observations	Refs
In vivo	*LMNB1*^*BAC*^ transgenic mice	Mice show cognitive impairment, age-dependent motor deficits, epilepsy, aberrant myelin formation, axonal degeneration and age-dependent demyelination	[34]
In vivo	PLP-*LMNB1*^Tg^ transgenic mice	Mice present aberrant myelin formation, axonal degeneration, epilepsy and age-dependent motor deficits and demyelination, and age-dependent degeneration of the spinal cord white matter	[34, 35]
Ex vivo	Patients’ fibroblasts	Cells displayed an altered nuclear morphology, altered mRNA splicing, inflammation, oxidative stress	[37–40]
Ex vivo	Patients’ skeletal muscle fibers	Cells displayed oxidative stress	[40]
Ex vivo	Patients’ blood cells	Cells displayed altered mRNA splicing	[37]
In vitro	Oligodendrocytes overexpressing *LMNB1*	Controversial observations	[34, 35, 38, 41]
In vitro	Astrocytes overexpressing *LMNB1*	Cells displayed altered morphology, aberrant cellular signaling, reduced viability, and induction of reactivity	[12, 15, 38, 41, 42]
In vitro	Neurons overexpressing *LMNB1*	Cells displayed important morphological alterations	[39, 41, 43]
In vitro	Fibroblasts overexpressing *LMNB1*	Cells displayed nuclear morphological alterations and inflammation	[38]

## Cellular Morphology Alterations in ADLD Models

Given the importance of Lamin B1 structural function in the nucleus, the effects of *LMNB1* overexpression on cell morphology represent an interesting topic. Understandably, being ADLD a disease that affects CNS, brain cells have been the major focus. Even though the studies that assessed the morphological consequences of *LMNB1* overexpression are very few, morphological abnormalities of various entities have been observed in all of them (Table 2).

**Table 2:** Cellular morphology alterations observed in ADLD models

Cell type	Observations	Experimental model	Refs
Oligodendrocytes	Misshaped nuclei; No nuclear alteration at the ultrastructural level	N20.1 murine cell line overexpressing *LMNB1*	[41]
		MO3.13 human cell line overexpressing *LMNB1*;	[38]
Astrocytes	Abnormal and folded nuclei, nuclear envelope in stacked membrane layers, short and beaded processes	Post-mortem ADLD astrocytes	[12, 15]
		SVGp12 human cell line overexpressing *LMNB1*;	[41]
		U87-MG human cell line overexpressing *LMNB1*	[38]
Neurons	Increased membrane surface, intranuclear aggregates, reduction of axonal length, increased nuclear rigidity, axonal disintegration, degradation of the axon-myelin unit	C17.2 murine cell line overexpressing *LMNB1*;	[41]
		Primary mouse cortical neurons overexpressing *LMNB1*;	[43]
		N2a murine cell line overexpressing *LMNB1*;	[39]
		PLP-FLAG-*LMNB1* transgenic mice;	[34]
		*LMNB1*^BAC^ transgenic mice	[34]
Fibroblasts	Stiff, misshaped nuclei	ADLD fibroblasts;	[38, 39]
		Primary dermal fibroblasts overexpressing *LMNB1*	[38]

### Oligodendrocytes

As oligodendrocytes are the CNS cell type responsible for myelin production and maintenance in the CNS, they have been amongst the first cells in which the effects of Lamin B1 over production were investigated. Confocal microscopy images obtained in a study on the effects of *LMNB1* ectopic overexpression on N20.1 murine oligodendrocytic cell line revealed that *LMNB1* overexpressing cells display misshaped nuclei [41]. On the contrary, a recent study on MO3.13 human oligodendrocytic cell line revealed at transmission electron microscopy (TEM) analysis that *LMNB1* overexpressing MO3.13 cells do not show any particular nuclear alteration at the ultrastructural level [38].

### Astrocytes

The presence of an altered astrocyte morphology has been first highlighted by the histopathological analysis of *post-mortem* ADLD brain tissue. This revealed astrocytes with significantly shortened and beaded cell processes [12, 15]. This evidence paved the way for the theory that a primary and relevant astrocyte pathology might also be present in ADLD. This theory is supported by the study by Lin and Fu (2009) that tested the effects of *LMNB1* overexpression also on the SVGp12 human astrocytic cell line, observing abnormal nuclear morphology in these cells as well [41], and by a study using U87-MG human astrocytic cell line. This last study revealed that the *LMNB1* overexpressing U87-MG cells display strong morphological alterations: immunofluorescence and TEM analyses showed deep alterations in the nuclear shape, with misshaped and folded nuclei and stacked membrane layers in the nuclear envelope [38].

### Neurons

The effects that Lamin B1 accumulation has on cellular structure have also been investigated in neurons. Ectopic overexpression of *LMNB1* in the C17.2 murine neural cell line revealed an increased surface area of the nuclear membrane and the presence of intranuclear aggregates [41]. *LMNB1* transient overexpression in primary mouse cortical neurons resulted in a significant reduction in axonal length, while dendritic trees seemed to not be affected [43]. Moreover, transiently *LMNB1* overexpressing murine neuronal N2a cells had increased nuclear rigidity [39]. Finally, in a transgenic mouse model in which the *LMNB1* overexpression was selectively targeted to oligodendrocytes, axonal disintegration and the degradation of the axon-myelin unit were observed [34].

### Fibroblasts

Being ADLD an autosomal dominant disease, *LMNB1* overexpression is present in every cell of the body [7]. For this reason, the analysis of primary skin fibroblasts from ADLD patients has become of particular interest for studying ADLD dynamics, given the accessibility of the samples. In concordance with the findings obtained on the examined CNS cells, ADLD skin fibroblast display increased nuclear stiffness [38, 39] and irregular, misshaped nuclei [32, 38, 44]. The same observations have been made on primary dermal fibroblasts transduced with *LMNB1* [38, 44].

## Cellular Function Alterations Involved in ADLD Models

Mechanical and structural alterations of the nuclear lamina have been observed in many pathologies [45]. This suggests that perturbations in the nuclear lamina meshwork have strong repercussions on cellular physiology. Notably, *LMNB1* is ubiquitously expressed in nearly every cell type. However, to date, its function is thought to be of particular importance in the CNS. This hypothesis is supported by studies performed on mouse models and on cell lines that demonstrated how Lamin B1 deficiency causes defects in brain development and neural cell function [43, 46, 47] and strengthened by the ADLD clinical phenotype i.e., CNS demyelination. Additionally, the reason behind the selective effect on CNS of Lamin B1 over production and specifically on demyelination is yet to be clarified. Several studies have addressed the effects that *LMNB1* overexpression has on the myelinating process and, more in general, on cellular function [35, 37, 38, 40–42]. Although the evidence obtained needs further confirmation, it appears that Lamin B1 accumulation has an impact on some important cellular processes (Table 3).

**Table 3:** Cellular function alterations observed in ADLD models

Cellular process	Cell type	Observations	Experimental model	Refs
Lipid synthesis	Oligodendrocytes	Downregulation of myelin-related genes, reduction in myelin lipid synthesis, reduction of Plp1 levels and capability of binding YY1 transcription factor	PLP-*LMNB1* transgenic mice	[34, 35]
Cell differentiation	Oligodendrocytes	Premature arrest of cells differentiation	Mouse primary glial cells overexpressing *LMNB1*	[41]
Chromatin organization	Oligodendrocytes	Increase in the repressive histone marks H3K9me3, H3K273me3; decrease in the acetylated histone marks AcH3 and AcH4	PLP-*LMNB1* transgenic mice	[35]
LIF production and LIF-R signaling	Astrocytes	Low LIF production and LIF-R expression, downregulation of PI3K p110α and γ, p-Akt, Raptor, and p-Stat3	U87-MG cell line overexpressing *LMNB1*	[38]
Cell viability and reactivity	Astrocytes	Reduction of cyclin D1 and Ki-67 levels, increase in p27, GFAP and vimentin; GSK3β inactivation	U87-MG cell line overexpressing *LMNB1*	[42]
mRNA splicing	Fibroblasts, blood cells	Overexpression of RAVER2 and consequent inhibition of PTB resulting in abnormal splicing of its targets	Skin fibroblasts and whole blood cells from ADLD patients	[37]
Oxidative stress response	Fibroblasts, skeletal muscle fibers	OCT-1 levels increase at the nuclear periphery and inability to re-localize in the nucleoplasm after H_2_O_2_ treatment; higher ROS production after H_2_O_2_ treatment	Skin fibroblasts and skeletal muscle fibers from ADLD patients	[38, 40]
Inflammation	Fibroblasts	Higher p-NF-kB and p-STAT4 levels	Skin fibroblasts from ADLD patients, primary fibroblasts overexpressing *LMNB1*	[38]

### Lipid Synthesis in Oligodendrocytes

A genetic study on FLAG-PLP*-LMNB1* transgenic mice revealed that out of the 12 genes that were found downregulated in the spinal cord, 9 belong to lipid synthesis pathways. Moreover, the expression of 8 of these 9 genes is known to be enriched in myelinating oligodendrocytes [35]. These results were confirmed by lipidomic analysis, revealing a significant reduction in myelin lipids in transgenic mice compared to wild-type animals [35]. Another study performed on the same mouse model observed the reduction of the myelin proteolipid protein (Plp1), a highly abundant mature myelin protein, in the mouse oligodendrocytes and the decreased capability of binding its transcription factor YY1 [34]. Lipid dysregulation is a hypothesis drawing much attention, given that the ADLD clinical phenotype is demyelination and that myelin is a substance of lipidic nature.

### Oligodendrocytes Differentiation

LMNB1 overexpression in mouse primary glial cells resulted in the premature arrest of oligodendrocytes differentiation [41]. This might be caused by the reduced transcription of myelin genes and by the nuclear sequestration of myelin proteins such as myelin basic protein and proteolipid protein [41]. Both processes appear to derive from Lamin B1 accumulation, but the mechanisms driving this process are yet to be identified.

### Chromatin Organization in Oligodendrocytes

The nuclear lamina plays an important role in chromatin positioning. Indeed, genes within lamina-associated domains usually exhibit repressive histone marks and a reduction in transcriptional activity [48]. Histone modifications and specifically histone methylation and acetylation play an important role in regulating oligodendrocyte function [49, 50]. A study performed on oligodendrocytes from PLP-FLAG-*LMNB1* mice observed an age-dependent increase in the repressive histone marks H3K9me3 and H3K273me3 in oligodendrocytes derived from both 3 and 13 months-old mice compared with wild-type cells [35]. Moreover, the analysis of acetylated histone marks AcH3 and AcH4, which are associated with transcriptionally active genes, revealed a significant reduction of these marks in oligodendrocytes derived from 13 months-old mice but not in oligodendrocytes derived from 3 months old mice. These results indicate that the overexpression of *LMNB1* is associated with age-dependent chromatin modifications that favor transcriptional repression in the oligodendrocytes of PLP-FLAG-*LMNB1* mice, therefore suggesting an important role of Lamin B1 levels in chromatin organization. Nevertheless, this aspect needs further elucidation.

### LIF Signaling in Astrocytes

Leukemia inhibitory factor (LIF) is a member of the interleukine-6 cytokine family [51]. LIF is a multifunctional factor: the cellular pathways activated by its receptor LIF-R are involved in cell survival, differentiation and, in CNS, myelin deposition, and maintenance [52, 53]. In particular, LIF is a factor produced by astrocytes that acts on LIF-R, expressed both by astrocytes and oligodendrocytes [54]. A recent work on U87-MG astrocytic cell line demonstrated that *LMNB1* overexpression alters the expression and the activity of signaling molecules downstream LIF-R that, along with its ligand LIF, were downregulated at both mRNA and protein levels [38]. Lamin B1 overexpression caused downregulation of the mediators of the PI3K pathway: the amounts of PI3K subunits p110α and γ were reduced as well as Akt phosphorylation and Raptor, a member of the mTORC1 complex. Moreover, the Stat3 pathway resulted downregulated [38]. LIF exogenous administration in *LMNB1* overexpressing cells resulted in an increase of PI3K p110α and γ, Raptor protein quantity, and Akt phosphorylation, but not Stat3 phosphorylation [38]. This study suggests that one of the causes behind ADLD’s demyelinating phenotype could be the impairment of LIF-R-related signaling pathways due to the inability of ADLD astrocytes to produce LIF. Nevertheless, the mechanism through which *LMNB1* overexpression affects LIF production is still to be understood. However, these results strengthen the hypothesis that astrocyte dysfunction plays a significant role in ADLD disease mechanisms by reducing the support to myelinating oligodendrocytes.

### Cell Viability and Reactivity in Astrocytes

Additional supporting evidence of the leading role of astrocytes in ADLD pathological mechanism was provided by studying the effects of *LMNB1* overexpression on the U87-MG cell line. Indeed, it was demonstrated how Lamin B1 accumulation causes the reduction of astrocyte viability and induces their reactive status [42]. Following *LMNB1* transduction, cells showed a marked decrease of cyclin D1 and of Ki-67 paralleled by an increase of p27, indicating a block in cell cycle progression and proliferation. Moreover, it was observed that *LMNB1* overexpression alters cell survival pathways by causing GSK3β inactivation, but not the upregulation of β-catenin targets, therefore resulting in a reduction in astrocyte survival. Finally, *LMNB1* overexpressing cells showed higher levels of cytotoxicity and apoptosis and displayed an increased immunoreactivity for Glial Fibrillary Acidic Protein (GFAP) and vimentin, both of which are well-characterized astrocytes reactivity markers [42]. Indeed, these results suggest that ADLD could be an astrocytopathy and, therefore, that demyelination might be caused by a primary astrocyte dysfunction. Nevertheless, this data still needs to be confirmed on patients’ cells and brain tissue, and the eventual role of astrocytes’ reactivity in ADLD pathology is yet to be clarified. However, the direct involvement of these cells in triggering the disease phenotype should be taken into consideration.

### Messenger-RNA Splicing in Fibroblasts and Blood Cells

The ribonucleoprotein PTB binding protein 2, referred to as RAVER2, is a regulator of the alternative splicing process. As its name suggests, it regulates the activity of the splicing repressor polypyrimidine tract binding protein (PTB) by inhibiting it [55]. Functional studies on mRNA extracted from skin fibroblasts and whole blood of ADLD patients demonstrated that RAVER2 expression is positively regulated by the levels of Lamin B1, being both proteins increased in ADLD cells. The upregulation of RAVER2 translates to an abnormal splicing pattern of several PTB-target genes, including the PLP1 gene that encodes for the most abundant mature myelin protein [37]. Therefore, these results indicate that demyelination in ADLD could be caused by a spliceopathy involving RAVER2 aberrant activity.

### Oxidative Stress Response in Fibroblasts

The Octamer Binding Transcription Factor 1 (OCT-1) regulates the expression of essential genes for the cellular response to oxidative stress [56]. It has been shown that Lamin B1 is able to bind OCT-1, recruiting it to the nuclear lamina and consequently reducing the binding with its target genes [57]. The study of OCT-1 in ADLD skin fibroblasts revealed that OCT-1 staining was significantly increased at the nuclear periphery compared to controls [40]. Moreover, in response to H_2_O_2_ treatment, OCT-1 re-localization to the nucleoplasm was significantly reduced in ADLD fibroblasts compared to controls [40]. This result suggests that ADLD cells have a higher susceptibility to oxidative stress than normal cells. This hypothesis is also supported by the data obtained in another study on ADLD skin fibroblasts: after H_2_O_2_ treatment, ADLD fibroblasts showed a higher reactive oxygen species (ROS) production compared to controls [38]. Since ROS levels have been shown to increase in the brain with age, it is possible that once a critical threshold is reached cellular damage is triggered [58]. Interestingly, in a model of Ataxia Telangiectasia, an autosomal-recessive disorder characterized by neurological defects [59], Lamin B1 levels have been observed to increase after treatment with H_2_O_2_ or L-buthionine sulfoximine (a compound that depletes the glutathione pool) [60]. Therefore, ROS-dependent cellular damage in the brain might be accelerated in ADLD due to higher ROS levels and the creation of a possible loop where ROS accumulation further increases Lamin B1 buildup. This scenario could also partially explain the age dependence of this pathology.

### Inflammation in Fibroblasts

Fibroblasts from ADLD patients and those from healthy donors transduced with *LMNB1* were found to display a higher activation of inflammatory pathways compared to controls: in western blot analysis, the amount of phosphorylated NF-kB and Stat4 was consistently higher in ADLD fibroblasts [38]. This evidence supports the hypothesis that ADLD cells are more subject to cellular stress than wild-type cells and therefore to cellular damage.

## Conclusion

Even though more than a decade has passed since the identification of the genetic cause of ADLD, the pathological mechanisms underlying this neurological disease have not been fully elucidated. It is clear that the increased amount of Lamin B1 leads to perturbations of inner nuclear membrane proteins, chromatin organization, and more in general, nuclear and cellular physiology. Nevertheless, how Lamin B1 accumulation causes these negative effects is still unclear. Even though the ADLD models developed to date have limitations, the evidence obtained from their study suggests two important considerations. First, *LMNB1* overexpression results in morphological alterations, especially at the nuclear level. These morphological alterations appear to be different from cell to cell, with cell types that are likely to be more sensitive to increased Lamin B1 levels (i.e., astrocytes) and cell types that seem to be more resistant (i.e., oligodendrocytes and neurons). Second, the morphological perturbations in these cells probably translate into functional alterations in some important cellular processes. In this case as well, the processes involved appear to be different depending on the cell type but are all leading to the common effect of causing myelin loss. Clearly, these hypotheses need further testing for confirmation. Moreover, it is important to put new effort into developing more faithful ADLD models. Under this light, focusing on patients’ cells could be the solution to overcome the problems that derive from animal and cellular models. Indeed, being finally able to uncover the dynamics that take place in this extremely complex and peculiar disease would not only give hope to the individuals affected by ADLD, who currently have no specific or curative therapy, that a treatment may be forthcoming, but could also be of major help in the study of other disorders that affect the CNS white matter.

## Data Availability

Not applicable.

## Abbreviations

ADLD: Autosomal dominant leukodystrophy
Akt: Protein kinase B
CNS: Central nervous system
GFAP: Glial fibrillary acidic protein
H_2_O_2_: Hydrogen peroxide
LIF: Leukemia inhibitory factor
LIF-R: Leukemia inhibitory factor receptor
MS: Multiple sclerosis
mTORC1: Mammalian target of rapamycin complex 1
NF-kB: Nuclear factor kappa B
OCT-1: Octamer binding transcription factor 1
PI3K: Phosphoinositide 3 kinase
PLP: Myelin proteolipid protein
PTB: Polypyrimidine tract binding protein
ROS: Reactive oxygen species
TEM: Transmission electron microscopy
YY1: Ying Yang 1
